# Socioeconomic disparities in sleep duration are associated with cortical thickness in children

**DOI:** 10.1002/brb3.2859

**Published:** 2022-12-27

**Authors:** Melissa Hansen, Katrina R. Simon, Jordan Strack, Xiaofu He, Kimberly G. Noble, Emily C. Merz

**Affiliations:** ^1^ Department of Psychology Colorado State University Fort Collins Colorado USA; ^2^ Department of Human Development Teachers College, Columbia University New York USA; ^3^ Department of Psychiatry Columbia University Irving Medical Center New York USA

**Keywords:** amygdala volume, basolateral amygdala, cortical thickness, family routines, sleep duration, socioeconomic status

## Abstract

**Introduction:**

Disrupted sleep has been consistently linked with lower academic achievement and worse mental health in children. Less is understood about sleep as a potential factor underlying socioeconomic differences in brain morphometry in children. The goals of this study were to investigate the associations among socioeconomic factors, sleep duration, and brain morphometry in children, and to examine the roles of the sleep environment and family routines in these associations.

**Methods:**

Participants were 5‐ to 9‐year‐old children from socioeconomically diverse families (*N* = 94; 61% female). Parents reported on children's weekday and weekend sleep durations, sleep environment, and family routines. High‐resolution, T1‐weighted structural magnetic resonance imaging (MRI) data were acquired. Analyses focused on cortical thickness, cortical surface area, and amygdala and hippocampal volume.

**Results:**

Results indicated that lower family income‐to‐needs ratio and parental education were significantly associated with shorter weekday sleep duration in children. Shorter weekday sleep duration was significantly associated with reduced thickness in the left middle temporal, right postcentral, and right superior frontal cortices and smaller basolateral but not centromedial amygdala volume. Family routines significantly mediated the associations of family income‐to‐needs ratio and parental education with weekday sleep duration in children.

**Conclusion:**

These results contribute to our understanding of sleep factors as proximal mechanisms through which socioeconomic context may alter neural development during childhood.

## Graphical abstract

In this study, we examined the associations among socioeconomic background, sleep duration, and brain structure in children and found that shorter sleep durations were linked to reduced cortical thickness in the left middle temporal, right postcentral, and right superior frontal regions. Additionally, shorter sleep durations were linked to reduced volume in the amygdala, specifically the basolateral amygdala. These findings are the first to point to socioeconomic disparities in sleep duration as a mechanistic pathway linking socioeconomic disadvantage with reduced cortical thickness and amygdala volume.

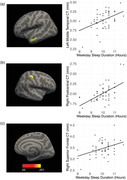

## 1 INTRODUCTION

Socioeconomic disadvantage, which is estimated to affect more than one in five children in the United States, has been consistently associated with lower cognitive development, academic achievement, and mental health in children (Bradley & Corwyn, 2002; McLoyd, 1998). At the neural level, research has frequently uncovered socioeconomic differences in brain structure in children (Farah, 2017), providing critical insights into neural mechanisms that inform interventions (Farah, 2018; Pollak & Wolfe, 2020a; Raizada & Kishiyama, 2010). Socioeconomic context is thought to represent a distal environmental factor that influences children's development through various proximal factors (Bronfenbrenner & Morris, 1998). Sleep duration is a potentially important proximal factor through which socioeconomic circumstances may impact brain structure in children. Insufficient sleep has been found to negatively impact mental health and academic achievement (Astill et al., 2012; Simon et al., 2020; Zhang et al., 2017) and is disproportionately found among children from socioeconomically disadvantaged environments (Buckhalt, 2011; Doane et al., 2019; Jarrin et al., 2014). However, the associations among socioeconomic factors, sleep duration, and brain structure in children remain poorly understood. As such, the main goal of this study was to examine the associations among socioeconomic factors, sleep duration, and brain structure in children.

### 1.1 Socioeconomic circumstances and sleep duration

Sleep duration, which refers to the amount or quantity of sleep, is one of the most frequently employed sleep measures. Children need certain amounts of sleep for healthy development and functioning, with the needed amount decreasing with age across childhood (Paruthi et al., 2016). Along with reduced sleep quality, reduced sleep duration is a core indicator of poor sleep or sleep disruption that has been associated with lower cognitive functioning, academic achievement (Dewald et al., 2010; Galvan, 2020; Short et al., 2018), and emotional and behavioral health (Astill et al., 2012; Biggs et al., 2011; Cheng et al., 2021; El‐Sheikh et al., 2020; Moore & Meltzer, 2008; Simon et al., 2020; Zhang et al., 2017). Indeed, insufficient sleep disrupts functioning in multiple cognitive and emotional domains, including memory consolidation, learning, processing speed, sustained attention, and cognitive and emotion regulation (Galvan, 2020; Krause et al., 2017; Riggins & Spencer, 2020; Sterpenich et al., 2007; Vriend et al., 2013; Yoo et al., 2007).

Although many children do not get the recommended amount of sleep for their age, children from socioeconomically disadvantaged environments are at particularly elevated risk for shorter sleep durations including insufficient amounts of sleep (Biggs et al., 2013; Buckhalt, 2011; Doane et al., 2019; El‐Sheikh et al., 2013; Jarrin et al., 2014). In a recent meta‐analysis, neighborhood‐level socioeconomic disadvantage was robustly associated with shorter sleep duration in children (Tomfohr‐Madsen et al., 2020). Additionally, associations between socioeconomic disadvantage and shorter sleep durations have been documented using both objective (e.g., actigraphy) and subjective measures of sleep duration (Doane et al., 2019; Jarrin et al., 2014).

### 1.2 Sleep duration and brain structure

Although sleep duration has been associated with brain structure in adults and adolescents (Galvan, 2020; Jalbrzikowski et al., 2021; Sung et al., 2020; Urrila et al., 2017), fewer studies have focused on younger children. Extant research has suggested significant associations between sleep duration and cortical and subcortical structure in children and adolescents (Dutil et al., 2018). For example, in one large‐scale study, shorter parent‐reported sleep durations were linked with lower cortical surface area (SA) and volume in frontal, temporal, and parietal cortical regions in 9‐ to 11‐year‐olds (Cheng et al., 2021). In other studies, greater parent‐reported total sleep disturbances (e.g., bedtime resistance, sleep onset delay, short sleep duration, sleep anxiety, nighttime awakenings, daytime sleepiness) were associated with smaller volumes and reduced cortical thickness (CT) in the dorsolateral prefrontal cortex at 7 years of age (Kocevska et al., 2017) and with reduced SA in the left middle temporal gyrus in 7‐ to 8‐year‐old children (Na et al., 2021).

Nonhuman animal studies have shown that experimentally induced sleep deprivation affects amygdala and hippocampal structure (Raven et al., 2018). In humans, studies of sleep duration and hippocampal volume in children and adolescents have yielded mixed findings. Shorter sleep durations have been significantly associated with both smaller (Taki et al., 2012) and larger hippocampal volume (Jalbrzikowski et al., 2021) in children and adolescents. Other studies have not found significant associations between sleep duration and hippocampal volume in children and adolescents (Cheng et al., 2021; Kocevska et al., 2017; Urrila et al., 2017). Hippocampal subfields include the cornu ammonis areas 1–4 (CA1–4), dentate gyrus, and subiculum (Merz et al., 2019; Teicher et al., 2012), which are cytoarchitecturally distinct and play distinct roles in learning and memory (Carlson et al., 2017; Evstratova & Tóth, 2014; Kesner, 2007). Sleep measures have been associated with hippocampal subfield volume in a few studies (Teicher et al., 2017). For example, in a study of 4‐ to 8‐year‐old children, longer sleep durations were associated with larger volumes in the CA2–4 and dentate gyrus subfields in younger but not older children (Riggins & Spencer, 2020).

Although functional magnetic resonance imaging (fMRI) studies have pointed to sleep‐related differences in amygdala activation in children and adolescents (Hehr et al., 2019; Reidy et al., 2016; Robinson et al., 2018), findings from the few studies of amygdala volume in children or adolescents have been mixed. In one study, shorter sleep durations were associated with larger amygdala volume in 9‐ to 25‐year‐olds (Jalbrzikowski et al., 2021). However, in another study, sleep duration was not found to be significantly associated with amygdala volume in 9‐ to 11‐year‐olds (Cheng et al., 2020). The amygdala includes the basolateral (BLA) and centromedial amygdala (CMA) subregions, which have distinct cellular architecture and connectivity patterns (Mosher et al., 2010; Rajbhandari et al., 2017; Skelly et al., 2020). Previous literature has suggested effects of early life stress on both the BLA and CMA (McEwen, 2007; Oshri et al., 2019).

In sum, many of the existing studies have focused on samples with wide age ranges or only included adolescents or adults. It is crucial to investigate the ways in which sleep duration may impact brain structure in younger children to understand the earlier emergence of these associations. In addition, research is needed to examine whether sleep duration has differential associations with hippocampal and amygdala subregional volumes.

### 1.3 Socioeconomic circumstances, sleep environment, and family routines

**FIGURE 1:**
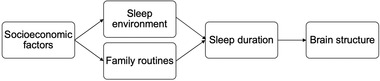
Mechanistic model in which socioeconomic factors are hypothesized to influence sleep environment quality and the frequency of family routines, which affect sleep duration and in turn brain structure in children

The sleep environment and family routines may represent specific mechanisms through which socioeconomic circumstances impact children's sleep duration, leading to differences in brain structure (see Figure 1). Socioeconomic disadvantage has been associated with lower quality sleep environments characterized by high noise levels, uncomfortable temperatures, excessive light, and crowded bedrooms (Bagley et al., 2015; Chung et al., 2014). Lower quality sleep environments have been shown to lead to shorter sleep durations and reduced sleep quality (Bagley et al., 2015; LeBourgeois et al., 2005).

Family routines may also partially explain the association between socioeconomic factors and sleep duration in children. Family routines refer to observable, repetitive behaviors involving family members that occur with predictable regularity during everyday family life (Boyce et al., 1983; Jensen et al., 1983; Sytsma et al., 2001). Socioeconomic disadvantage has been associated with less consistent family routines and greater environmental unpredictability (Evans, 2004; Evans & Kim, 2013), which may increase children's stress and interfere with their sleep (Philbrook et al., 2020). Yet, to date, we have an incomplete picture of the associations among socioeconomic circumstances, family routines, sleep duration, and brain structure in children.

Collectively, in separate literatures, socioeconomic disadvantage has been associated with shorter sleep durations (Doane et al., 2019; Jarrin et al., 2014), and sleep duration has been associated with brain structure, with many magnetic resonance imaging (MRI) studies of sleep focused on adolescents, adults, or clinical samples (e.g., insomnia, sleep apnea) (Cheng et al., 2021; Jalbrzikowski et al., 2021; Philby et al., 2017). However, the associations among socioeconomic context, sleep duration, and brain structure in typically developing children are not well understood. In addition, many previous studies have used measures of sleep duration that did not distinguish between weekday and weekend sleep duration, which can be different for multiple reasons (e.g., children attend school on weekdays) (Biggs et al., 2013; Falbe et al., 2015; Taki et al., 2012; Urrila et al., 2017). For instance, some studies estimated sleep duration by averaging across the 7 days of the week; some focused only on weekdays or weekends; and others used self‐ or parent‐reported “usual” sleep duration without specifying which day of the week (Gupta et al., 2018). Understanding the associations among socioeconomic factors, sleep duration, and brain structure in children would have important mechanistic and applied implications. Sleep duration may represent a modifiable factor that could be targeted in interventions to reduce socioeconomic differences in brain development and in turn cognitive and mental health outcomes.

### 1.4 Current study

Following from our hypothesized mechanistic model (see Figure 1), the main goals of this study were to investigate (1) the associations among socioeconomic factors (family income‐to‐needs ratio, parental education), sleep duration, and brain structure and (2) the roles of the sleep environment and family routines in associations between socioeconomic factors and sleep duration in children. Participants were 5‐ to 9‐year‐old children from socioeconomically diverse families. Parents reported on children's weekday and weekend sleep durations, sleep environment, and family routines. High‐resolution, T1‐weighted MRI data were acquired, and analyses focused on CT, SA, and amygdala and hippocampal volume.

We hypothesized that shorter sleep duration would be associated with reduced CT and/or SA and reduced hippocampal and amygdala volume in children. We also predicted that sleep duration would mediate associations between socioeconomic factors and brain structure (see Figure S1). If sleep duration was associated with total volume in the hippocampus and/or amygdala, we then examined whether the associations were attributable to specific subregions of these structures. Based on previous research (McEwen, 2007; Oshri et al., 2019; Riggins & Spencer, 2020; Teicher et al., 2017), we tentatively hypothesized that sleep duration would be associated with CA3 and dentate gyrus hippocampal subfield volume and both BLA and CMA volume. Finally, we expected that socioeconomic disadvantage would be associated with a lower quality sleep environment and fewer family routines, which would in turn be associated with shorter sleep duration in children (see Figure S2).

## 2 METHODS

### 2.1 Participants

Participants were recruited through posted flyers and local community events in New York, NY. Parents and their children participated in this study, and the age of the children ranged from 5 to 9 years (*N* = 94; 61% female). Families were included if they were primarily English speakers, and if the children were born from a singleton pregnancy and had no history of premature birth (<37 gestational weeks at birth) or medical or psychiatric problems. Children with MRI contraindications were also excluded. Parental educational attainment ranged from 6.50 to 20.00 years, and family income‐to‐needs ratio ranged from 0.17 to 15.21. Family income ranged from $2880 to $350,000 (see Table 1 for sample characteristics).

**TABLE 1:** Descriptive statistics for sample characteristics (*N* = 94)

	Mean	SD
Child age (years)	7.03	1.29
Family income‐to‐needs ratio	2.68	2.79
Family income (U.S. dollars)	65,537.23	69,277.75
Parental education (years)	14.14	2.64
Parental anxiety symptoms	7.60	7.02
Parental depression symptoms	5.79	4.92

	%	n
Child sex (female)	60.64	57
Child race/ethnicity		
African American, non‐Hispanic/Latinx	30.85	29
Hispanic/Latinx	50.00	47
European American, non‐Hispanic/Latinx	13.83	13
Other	5.32	5
Family income below U.S. poverty line	29.79	28

### 2.2 Procedure

Parents and children visited the lab on two separate occasions within 1 month. During the first study visit, parents completed questionnaires regarding socioeconomic factors, sleep duration and environment, and family routines. Additionally, most participants were offered the opportunity to participate in the MRI portion of the study, and the children completed a mock MRI scan to gain familiarity with the MRI environment (Merz et al., 2019, 2020). During the second study visit, children participated in an MRI scanning session that included a T1‐weighted structural sequence.

### 2.3 Measures

#### 2.3.1 Socioeconomic factors

Data on family income, parental education, and the number of people in the household were collected. Parental educational attainment was calculated as the number of years of education averaged across parents. Family income‐to‐needs ratio was calculated by dividing household income by the U.S. federal poverty line for the number of people in the household (for the year they participated in the study) (Pollak & Wolfe, 2020b). Family income‐to‐needs ratio was logarithmically transformed to correct for positive skew. Parental education and family income‐to‐needs ratio were significantly correlated (*r* = .68, *p* < .001). Family income‐to‐needs ratio and parental education were examined separately as they may represent distinct aspects of children's environments that relate differentially to their development (Duncan et al., 2012).

#### 2.3.2 Sleep duration

Parents reported on their children's bedtimes and wake‐up times for a typical weekday and weekend day in the past 2 weeks. Questions included “What is your child's weekday bedtime?” and “When does your child wake up on weekdays?” Child sleep duration was calculated separately for weekdays and weekends by computing the amount of time between bedtime and wake‐up time, similar to previous studies (Gupta et al., 2018; Urrila et al., 2017).

#### 2.3.3 Sleep environment

Child sleep environment was measured through parent report on six questionnaire items adapted from the National Sleep Foundation's six criteria for a “good sleep environment” (Mindell et al., 2009). These items assessed the extent to which sleep environments were characterized by minimal sound, minimal light, optimal temperature, a comfortable sleeping surface, nondisruptive bedroom partners (e.g., no snoring or noisy siblings), and no technological activity (e.g., cell phones, computers, televisions) immediately before bedtime. Parents responded on a 5‐point scale ranging from 1 (*strongly disagree*) to 5 (*strongly agree*). The possible total scores on the sleep environment measure ranged from 0 to 30, with 0 being low and 30 being high.

#### 2.3.4 Family routines

Parents also completed the Family Routines Inventory (Jensen et al., 1983), a 28‐item measure of the regularity of specific family routines in any given week. Parents were asked to rate the frequency of certain routines in their family (e.g., “Children do the same things every morning as soon as they wake up,” “Family has certain ‘family time’ each week when they do things together at home”). Each item is rated on a 4‐point scale, with 0 = *almost never*, 1 = *one to two times per week*, 2 = *three to five times per week*, and 3 = *always–every day*. Higher scores on this measure indicate greater regularity of family routines. This scale has demonstrated adequate reliability and validity (Jensen et al., 1983; Sytsma et al., 2001). In the present study, one item that assumed the presence of a younger sibling was omitted from scoring. Internal consistency was acceptable in the current sample (Cronbach's *α* = .72).

#### 2.3.5 Parental anxiety and depression symptoms

Parental depressive symptoms were measured using the nine‐item Patient Health Questionnaire (PHQ‐9; Kroenke et al., 2001), a self‐report measure of depression based on the Diagnostic and Statistical Manual (DSM) criteria for major depressive disorder. Parents indicated how often in the past 2 weeks they were bothered by depressive symptoms using a 4‐point scale ranging from 0 (*not at all*) to 3 (*nearly every day*). Responses were then summed to create a total score, with higher total scores indicating greater depressive symptoms (*α* = .84). The PHQ‐9 has well‐established internal consistency, test–retest reliability, and validity (Kroenke et al., 2001; Lee et al., 2007).

Parental anxiety symptoms were measured using the Beck Anxiety Inventory (BAI; Beck & Steer, 1990), a 21‐item self‐report measure of physiological and cognitive anxiety symptoms. Participants are asked to indicate how often in the past week they were bothered by anxiety symptoms using a 4‐point scale ranging from 0 (*not at all*) to 3 (*severely/could barely stand it*). Participant responses are then summed to create a total score with higher scores indicating greater anxiety symptoms (*α* = .91). The BAI has strong internal consistency, test–retest reliability, and concurrent validity (Beck & Steer, 1991). A parental anxiety/depression composite score was created to use as a covariate in analyses by standardizing and averaging the PHQ‐9 and BAI scores.

### 2.4 Imaging acquisition and processing

MRI data were acquired on a 3‐Tesla General Electric (GE) MR750 scanner with a 32‐channel head coil using a high‐resolution T1‐weighted fast spoiled gradient echo sequence (repetition time [TR] = 7.1 ms; inversion time [TI] = 500 ms; flip angle = 11 degrees; 176 sagittal slices; 1.0 mm slice thickness; field of view [FOV] = 25 cm; in‐plane resolution = 1.0 × 1.0 mm). In total, 66 children were scanned. All images were visually inspected for motion artifacts and ghosting. Consequently, 15 children's T1‐weighted MRI data were excluded from analyses, resulting in a sample size of 51 children with usable MRI data (Merz et al., 2019, 2020). There was no manual editing of data that were deemed eligible for inclusion, similar to previous studies (Noble et al., 2015). The MRI subsample (*n* = 51) did not differ significantly in socioeconomic background from those without MRI data.

Images were processed using standard automated procedures in FreeSurfer 6.0 (http://surfer.nmr.mgh.harvard.edu/). These procedures included removal of nonbrain tissue, image intensity normalization, and construction of white/gray matter and gray matter/cerebrospinal fluid boundaries (Dale et al., 1999; Fischl & Dale, 2000). Automatic parcellation and segmentation in FreeSurfer 6.0 was conducted (Dale et al., 1999; Fischl & Dale, 2000; Fischl et al., 2004). The Desikan–Killiany atlas (Desikan et al., 2006) was used to compute CT and SA in anatomically defined regions, and these data were used as needed following the vertex‐wise analyses.

Volumes of the amygdala subnuclei were segmented using an automatic algorithm in FreeSurfer 6.0 (Saygin et al., 2017). Volumetric measurements were obtained for the following subnuclei in each hemisphere: lateral nucleus, basal nucleus, accessory basal nucleus, anterior amygdaloid area, central nucleus, medial nucleus, cortical nucleus, corticoamygdaloid transition area, and paralaminar nucleus. BLA and CMA volumes were calculated from those segmentations to use in analyses. BLA volume was calculated by adding the volumes of the basal, lateral, and basal accessory nuclei, averaged across right and left hemispheres (LeDoux, 2000; Manassero et al., 2018). CMA volume was calculated by adding the volumes of the central and medial nuclei, averaged across right and left hemispheres (Oshri et al., 2019).

Hippocampal subfield segmentation was conducted using the automated algorithm available in FreeSurfer 6.0 (Iglesias et al., 2015). Hippocampal subfields that were segmented included the CA1, CA2/3 (combined in the atlas because of indistinguishable MRI contrast), CA4, granule cell layer of the dentate gyrus, and subiculum. Dentate gyrus volume was calculated by summing the volumes of the CA4 and granule cell layer subfields. All subcortical segmentations passed visual inspection for major errors.

### 2.5 Statistical analyses

Multiple linear regression analyses were conducted in R (version 4.1.1) to examine associations among socioeconomic factors, sleep duration, and amygdala and hippocampal volume. Covariates included child age, sex, parental education, and total brain volume. Race/ethnicity was not included as a covariate as it was unrelated to sleep duration. All tests were two tailed (*α* = .05). To account for multiple comparisons, false discovery rate (FDR) corrections were applied to analyses using the p.adjust function in R (Jafari & Ansari‐Pour, 2019).

Whole‐cortex, vertex‐wise multiple linear regression analyses were employed to examine associations of sleep duration with CT and SA. FreeSurfer's Query, Design, Estimate, Contrast (QDEC) surface‐based analysis tool was used. These analyses were conducted using a 10‐mm full‐width half‐maximum smoothing kernel and cluster‐wise correction for multiple comparisons. Cluster‐wise *p*‐value thresholds were set to .05, and the vertex‐wise threshold was set to .001 (Greve & Fischl, 2018). The two‐hemisphere correction was also applied to these analyses. All analyses controlled for child age, sex, parental education, and total brain volume.

#### 2.5.1 Mediation model 1

Analyses were conducted to examine the extent to which sleep duration may mediate associations between socioeconomic factors and brain structure (see Figure S1). Standard mediation analyses were performed using the “mediation” package in R software (Tingley et al., 2014). First, two regression models were specified: the mediator model for the conditional distribution of the mediator (e.g., sleep duration) given the independent variable (e.g., parental education, family income‐to‐needs ratio) and the outcome model for the conditional distribution of the outcome (e.g., brain structure) given the independent variable and the mediator. Subsequently, the outputs of these two regression models served as the main inputs to the “mediate” function that computes the direct, indirect, and total effects. The significance of the mediation was estimated by the nonparametric bootstrap approach (with 10,000 random samplings).

For any sleep duration measure (weekday or weekend) found to be associated with both a socioeconomic factor (family income‐to‐needs ratio, parental education) and a brain structural outcome (regional CT or SA, amygdala or hippocampal volume), analyses were conducted to examine whether that sleep duration measure mediated the association between socioeconomic context and the brain structural outcome (MacKinnon et al., 2002).

Rather than biasing the mediation analyses by extracting and using data from the vertex‐wise analyses (Kriegeskorte et al., 2009), CT/SA in anatomically defined cortical regions (Desikan et al., 2006) were used in these mediation analyses. Specifically, if sleep duration was associated with regional CT/SA in the vertex‐wise analyses, we used CT/SA in anatomically defined regions corresponding to the regions found to be significant in vertex‐wise analyses.

#### 2.5.2 Mediation model 2

These statistical procedures were repeated to examine whether sleep environment and family routines jointly or individually mediated the associations between socioeconomic factors and children's sleep duration (see Figure S2). In these analyses, the mediators were sleep environment and family routines; the independent variables were socioeconomic factors (parental education, family income‐to‐needs ratio); and the outcome was sleep duration (weekday and/or weekend). Similar to the analyses described above, if family routines and/or sleep environment were significantly associated with both a socioeconomic factor and sleep duration, then mediation analyses were conducted to test one or both as mediators (MacKinnon et al., 2002). If both met these criteria, then we used a multiple mediation model. If only one (family routines or sleep environment) met the criteria, then we tested a single mediator model.

## 3 RESULTS

### 3.1 Descriptive statistics

Descriptive statistics are provided in Table 2. The recommended amount of sleep for 5‐year‐olds is 10–13 h and for 6‐ to 9‐year‐olds is 9–12 h per night (Paruthi et al., 2016). On average, the children in our sample were within the recommended range of sleep duration for both weekdays and weekends. Yet, weekday sleep duration ranged from 8 to 12.5 h per night, and weekend sleep duration ranged from 7.5 to 13.5 h per night. Fourteen of the 94 children in our sample (six of the 5‐year‐olds and eight of the 6‐ to 9‐year‐olds) had less than the recommended minimum amount of sleep on either weekends or weekdays. Zero‐order correlations between socioeconomic factors, sleep duration and environment, family routines, and amygdala and hippocampal volumes are provided in Table S1.

**TABLE 2:** Descriptive statistics for sleep duration and environment, family routines, and amygdala and hippocampal volume

	Mean	SD
Weekday sleep duration (hours)	10.10	0.79
Weekend sleep duration (hours)	10.23	1.28
Sleep environment	22.20	4.11
Family routines	57.42	9.54
Amygdala volume (mm^3^)	2820.81	371.91
Hippocampal volume (mm^3^)	3145.65	311.86

Approximately 30% of families in the sample had their sleep data collected during the summer. Time of year (summer vs. school year) was not associated with weekday (*β* = –0.14, *p* = .50) or weekend sleep duration (*β* = –0.19, *p* = .52) while controlling for child age and sex.

### 3.2 Socioeconomic factors and sleep duration

Lower parental education (*β* = 0.11, *p* = .005) and family income‐to‐needs ratio (*β* = 0.47, *p* = .03) were significantly associated with shorter weekday sleep duration in children (see Figure S3). Neither socioeconomic factor was significantly associated with weekend sleep duration.

### 3.3 Sleep duration and brain structure

#### 3.3.1 CT and SA

**FIGURE 2:**
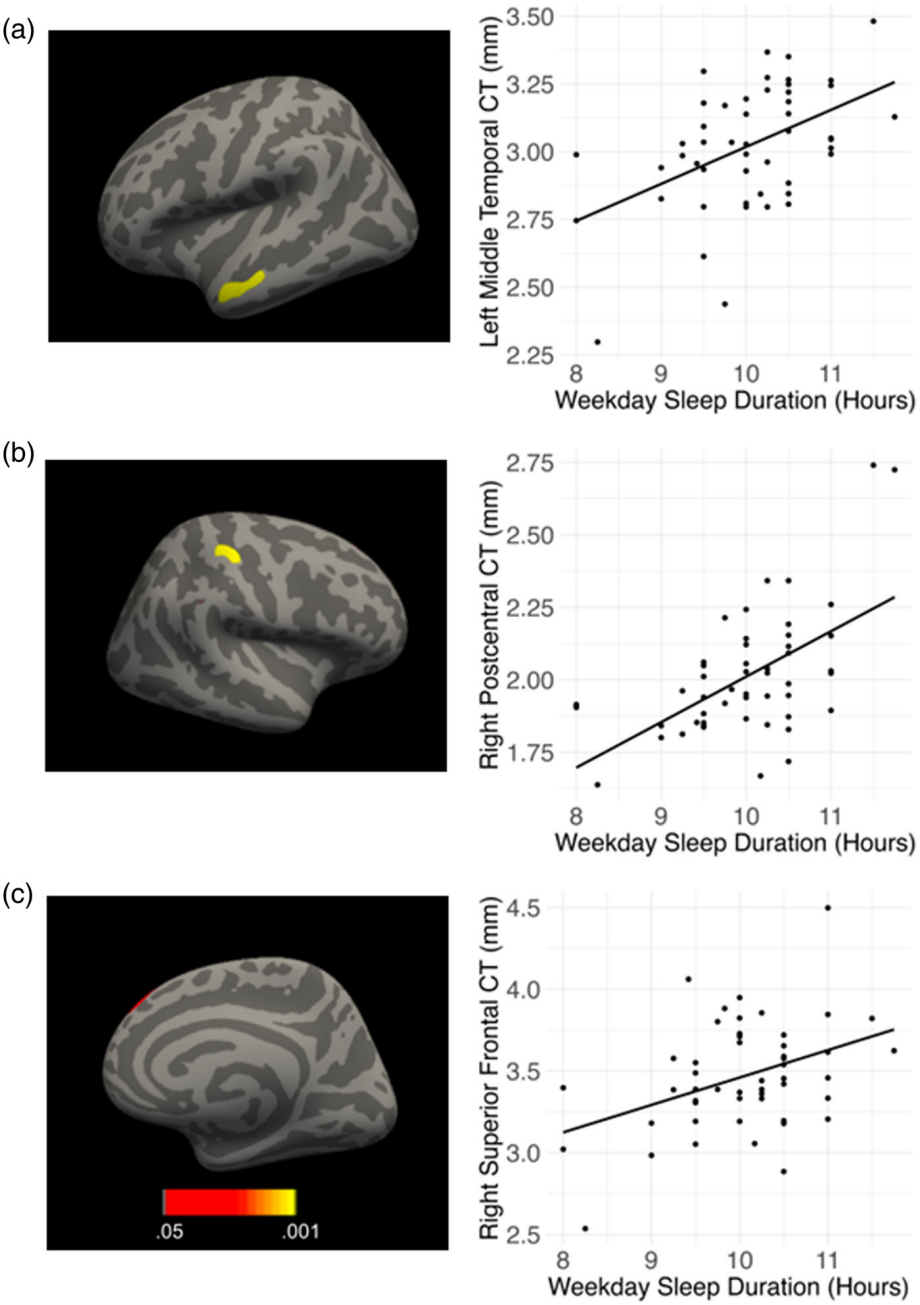
Longer weekday sleep duration was significantly associated with greater cortical thickness (CT) in the (a) left middle temporal, (b) right postcentral, and (c) right superior frontal gyri in children. Results are shown on the inflated surface. Colors represent *p*‐values.

Shorter weekday sleep duration was significantly associated with reduced CT in the left middle temporal gyrus, right superior frontal gyrus, and right postcentral gyrus (see Table 3 and Figure 2). Weekday sleep duration was not significantly associated with SA. Weekend sleep duration was not significantly associated with CT or SA.

**TABLE 3:** Associations between weekday sleep duration and cortical thickness (CT)

		MNI305 peak coordinates					
Cluster #	Label	x	y	z	Cluster size (mm^2^)	Number of vertices	p‐value
1	Left middle temporal gyrus	–49.8	–3.6	–24.8	404.45	594	.0002
2	Right postcentral gyrus	41.5	–21.1	47.9	311.71	585	.001
3	Right superior frontal gyrus	7.7	50.8	38.2	214.85	426	.023

Abbreviation: MNI, Montreal Neurological Institute.

#### 3.3.2 Amygdala and hippocampus

**FIGURE 3:**
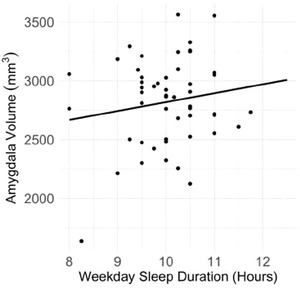
Longer weekday sleep duration was significantly associated with larger amygdala volume in children

Shorter weekday sleep duration was significantly associated with reduced amygdala volume (*β* = 1.74, FDR‐corrected *p* = .002) (see Figure 3). When examining amygdala subregional volumes, shorter weekday sleep duration was associated with reduced BLA (*β* = 0.49, FDR‐corrected *p* = .01) but not CMA volume. Weekend sleep duration was not significantly associated with amygdala volume, and weekday and weekend sleep durations were not related to hippocampal volume.

### 3.4 Socioeconomic factors, sleep duration, and brain structure

**FIGURE 4:**
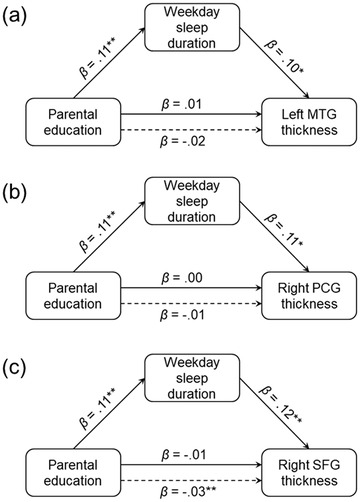
Parental education was indirectly associated with cortical thickness (CT) in the (a) left middle temporal gyrus (MTG), (b) right postcentral gyrus (PCG), and (c) right superior frontal gyrus (SFG) via weekday sleep duration in children. The solid line between parental education and CT represents the total effect (c path), while the dotted line represents the direct effect after accounting for the indirect effect (cʹ path). *p < .05, **p < .01

Lower parental education was significantly indirectly associated with reduced CT in the left middle temporal gyrus (*ab* = .01 *p* = .033), right postcentral gyrus (*ab* = .01, *p* = .004), and right superior frontal gyrus (*ab* = .02, *p* = .007) via shorter weekday sleep duration in children (see Figure 4). Weekday sleep duration did not mediate the association of socioeconomic factors with amygdala or BLA volume. Additionally, weekday sleep duration did not mediate associations between family income‐to‐needs ratio and CT. To rule out alternative interpretations, mediation models were run in which CT was examined as a mediator of associations between socioeconomic factors and sleep duration in children. These alternative models did not yield significant indirect effects.

### 3.5 Socioeconomic factors, family routines and sleep environment, and sleep duration

Lower parental education (*β* = 0.50, *p* = .002) and family income‐to‐needs ratio (*β* = 0.03, *p* = .01) were significantly associated with a lower quality sleep environment in children (see Figure S4). Similarly, lower parental education (*β* = 1.50, *p* = .0007) and family income‐to‐needs ratio (*β* = 8.30, *p* = .01) were significantly associated with less frequent family routines. Less frequent family routines were significantly associated with shorter weekday sleep duration (*β* = 0.03, *p* = .00005; see Figure S5), but sleep environment was not significantly associated with weekday sleep duration. Thus, we tested whether family routines mediated the association between socioeconomic factors and weekday sleep duration in children. Family routines significantly mediated the association between socioeconomic factors and weekday sleep duration in children (*ab* = .04, *p* < .001). Socioeconomic disadvantage was significantly associated with fewer family routines, which in turn significantly associated with shorter weekday sleep duration in children (see Figure S6).

### 3.6 Sensitivity analyses

Follow‐up analyses were conducted to examine associations of weekday sleep duration with CT in the left middle temporal gyrus, right postcentral gyrus, and right superior frontal gyrus and amygdala and BLA volume while additionally controlling for parental anxiety and depression symptoms. These analyses were conducted to account for any confounding impact that parental internalizing symptoms could have on parent reports of children's sleep durations, following previous research (Mulder et al., 2019). Associations of weekday sleep duration with CT in the left middle temporal gyrus (*β* = 0.11, *p* = .009), right postcentral gyrus (*β* = 0.10, *p* = .007), right superior frontal gyrus (*β* = 0.11, *p* = .01), amygdala volume (*β* = 0.17, *p* = .002), and BLA volume (*β* = 0.46, *p* = .045) remained significant.

## 4 DISCUSSION

The goals of this study were to investigate (1) the associations among socioeconomic factors, sleep duration, and brain structure in children and (2) the role of the sleep environment and family routines in socioeconomic differences in children's sleep duration (see Figure 1). Results indicated that socioeconomic disadvantage was significantly associated with shorter weekday sleep duration in children, replicating previous research (Doane et al., 2019; El‐Sheikh et al., 2013; Jarrin et al., 2014; Tomfohr‐Madsen et al., 2020). Shorter weekday sleep duration was significantly associated with reduced thickness in temporal, frontal, and parietal cortical regions and smaller amygdala volume, particularly in the BLA subregion. Parental education was significantly indirectly associated with CT via weekday sleep duration. Additionally, less frequent family routines mediated the association between socioeconomic disadvantage and shorter weekday sleep duration in children.

### 4.1 Sleep duration is associated with CT in children

Shorter weekday sleep duration was associated with reduced thickness in the left middle temporal gyrus, right superior frontal gyrus, and right postcentral gyrus in children. These results are consistent with previous findings linking sleep disruption, including shorter sleep durations, with reduced gray matter in frontal, temporal, and parietal regions in children and adolescents (Cheng et al., 2021; Isaiah et al., 2021; Jalbrzikowski et al., 2021; Kocevska et al., 2017; Na et al., 2021; Sung et al., 2020; Urrila et al., 2017). The left middle temporal gyrus plays a critical role in language processing, including word processing and semantics (Papeo et al., 2019). The right superior frontal gyrus has been associated with inhibitory control and motor functioning (Hu et al., 2016). The right postcentral gyrus, part of the primary somatosensory cortex, is associated with processing somatosensory input (Borich et al., 2015). Sleep‐related structural differences in these cortical regions may partially explain the well‐established associations of sleep duration with academic achievement and mental health in children (Astill et al., 2012; Galvan, 2020; Simon et al., 2020). Multiple cellular mechanisms may underlie the associations between shorter sleep duration and reduced CT, including dendritic remodeling and synaptic pruning (Raven et al., 2018).

Some previous studies have focused on cortical volume (Sung et al., 2020; Urrila et al., 2017), a composite of SA and CT, which have been documented as genetically and developmentally distinct (Panizzon et al., 2009; Raznahan et al., 2011). In other studies, SA and CT have been analyzed separately (Cheng et al., 2021; Isaiah et al., 2021; Jalbrzikowski et al., 2021; Kocevska et al., 2017; Na et al., 2021). In the current study, sleep duration was significantly associated with CT but not SA, consistent with some previous studies (Isaiah et al., 2021; Jalbrzikowski et al., 2021; Kocevska et al., 2017) but not others (Cheng et al., 2020; Na et al., 2021). More work is needed to understand the differential associations of sleep duration with CT and SA in children. Furthermore, longitudinal studies of CT and SA development are needed. In typical development, CT decreases during middle childhood and adolescence (Raznahan et al., 2011). Longitudinal research is needed to examine children's sleep duration in relation to their trajectories of cortical thinning across time and their cognitive and mental health outcomes.

### 4.2 Socioeconomic disparities in sleep duration are associated with CT in children

Lower parental education was significantly indirectly associated with reduced CT in these regions via shorter weekday sleep duration in children. Thus, socioeconomic disadvantage may lead to reductions in children's sleep duration, which in turn are associated with decreased regional CT. Socioeconomic context impacts many aspects of children's proximal environments that could affect their sleep, such as stress exposure, noise/crowding, and air pollution (Bronfenbrenner & Morris, 1998; Evans, 2004; Farah, 2017). Findings from our study suggest that less frequent family routines may partially explain why socioeconomic disadvantage may interfere with children's sleep. Specifically, lower family income‐to‐needs ratio and parental education were significantly associated with fewer family routines, which in turn were associated with reduced weekday sleep durations in children. These findings are consistent with research showing that socioeconomic disadvantage is associated with more chaotic and unpredictable home environments and fewer family routines (Evans, 2004; Evans & Kim, 2013), which can interfere with sleep duration and quality in children and adolescents (Hale et al., 2009; Philbrook et al., 2020; Yoo et al., 2010). Exposure to fewer family routines and greater environmental unpredictability may have increased children's exposure to stress (Evans, 2004; Evans & Kim, 2013), interfering with their sleep (Bagley et al., 2015).

These results for family routines could also be due to variability in bedtime routines, as consistent bedtime routines are important to children's sleep duration and quality (Buxton et al., 2015; Lee et al., 2019). Future studies should examine the frequency of bedtime routines in relation to sleep duration and brain structure in children (Henderson & Jordan, 2010). Reasons for reduced consistency of family routines likely vary across families. Determining the causes of inconsistent family routines through an evaluation would be an important first step to identifying an effective intervention. Although socioeconomic disadvantage was significantly associated with a lower quality sleep environment, the sleep environment did not mediate socioeconomic differences in children's sleep duration.

### 4.3 Sleep duration is associated with basolateral amygdala volume in children

Shorter weekday sleep duration was also associated with smaller amygdala volume, consistent with fMRI studies linking disrupted sleep with altered amygdala activation in children and adolescents (Reidy et al., 2016; Robinson et al., 2018). In the few structural MRI studies, findings for associations between sleep duration and amygdala volume have been mixed (Cheng et al., 2020; Jalbrzikowski et al., 2021). Results from the current study suggest that these inconsistencies may be partially due to sleep duration affecting some amygdala subnuclei more than others. More specifically, shorter weekday sleep duration was significantly associated with smaller BLA but not CMA volume. The BLA has been shown to be susceptible to early life stress (Oshri et al., 2019) and is associated with the regulatory response to emotionally salient environmental stimuli (Rajbhandari et al., 2017; Skelly et al., 2020). Sleep‐duration‐related differences in BLA volume may partially underlie the effects of sleep sufficiency on emotion processing and regulation, including fear conditioning and extinction (Simon et al., 2020). Shorter sleep durations could also cause increased stress or anxiety symptoms, which could lead to reductions in BLA volume.

Sleep duration was not found to be significantly associated with hippocampal volume. Previous studies of sleep and hippocampal volume in children and adolescents have yielded mixed results (Cheng et al., 2020; Kocevska et al., 2017; Taki et al., 2012; Urrila et al., 2017). It is possible that differences in hippocampal volume may be more sensitive to other sleep measures, such as indices of sleep quality or disturbances.

In contrast to weekday sleep duration, weekend sleep duration was not significantly associated with socioeconomic factors or brain structure in children. Previous studies have shown that weekday and weekend sleep parameters have differential associations with brain structure and mental health in children and adolescents (Taki et al., 2012; Urrila et al., 2017; Zhang et al., 2017). It is possible that insufficient sleep and the accumulation of sleep debt during the week are especially sensitive to socioeconomic context and brain structure. Also, reduced weekday sleep duration in children from socioeconomically disadvantaged families could be due in part to later bedtimes (Biggs et al., 2013). If this is the case, it could partially explain why socioeconomic factors were not associated with weekend sleep duration in children.

Taken together, these findings suggest that targeting children's sleep sufficiency in interventions may be an effective way of supporting healthy brain development and in turn cognitive development and mental health. Some research suggests that interventions that promote healthy sleep hygiene may lead to increases in sleep duration for children from socioeconomically disadvantaged families (Mindell et al., 2016). Sleep patterns and sufficiency can often become problematic during adolescence (Galvan, 2020). The results of this study suggest that interventions that start targeting sleep sufficiency during childhood, prior to adolescence, may be more effective than those that start later.

This study had many strengths including the use of a sample recruited to be socioeconomically diverse and thus well equipped to be used for research questions focused on socioeconomic context. In addition, this study focused on children younger (5–9 years of age) than most of the samples in previous studies. Rigorous MRI and statistical methods were also employed in this research, with comprehensive quality control of the MRI data. Several limitations should be taken into account when interpreting these findings. First, the cross‐sectional and correlational design of this study precludes making inferences about directionality and causality of the associations. Associations between sleep duration and brain structure in children may be bidirectional. Second, sleep duration was measured via parent report of children's bedtimes and wake‐up times, which may not precisely reflect time spent asleep. The strengths of this approach were that sleep was measured in children's natural environments and parents were asked about the past 2 weeks, increasing ecological validity. In addition, our findings remained significant after additionally controlling for parental anxiety and depression symptoms, which may confound parent reports of children's sleep (Mulder et al., 2019). Nonetheless, future studies should replicate these findings using objective measures of sleep duration such as actigraphy (Sadeh, 2015). Third, mediation questions are best examined using longitudinal data (Cole & Maxwell, 2003; Maxwell & Cole, 2007), which were not available as part of the data set used in this study. Fourth, eight children had their MRI data collected more than 1 month after their sleep data were collected. Associations between sleep duration and brain structure remained significant after controlling for the time between sessions.

In this study, socioeconomic disadvantage was associated with shorter sleep duration in children. Shorter sleep duration was associated with reduced CT in frontal, temporal, and parietal regions involved in language, inhibitory control, and sensorimotor processing. Reduced sleep duration was found to be a pathway through which socioeconomic disadvantage may lead to decreased CT in children. Promoting healthy sleep practices may support brain development in children from socioeconomically disadvantaged families.

## FUNDING INFORMATION

This study was supported by the National Center for Advancing Translational Sciences, National Institutes of Health, through grant numbers UL1TR001873 and UL1RR024156. Additional funding was provided by the Gertrude H. Sergievsky Center, Columbia University Medical Center; Teachers College, Columbia University; and a National Institute of Mental Health training grant (T32MH13043).

## CONFLICT OF INTEREST

The authors declare no conflict of interest.

## Supporting information

Table S1. Zero‐order correlations
Figure S1. Mediation model in which sleep duration was hypothesized to mediate associations between socioeconomic factors and brain structure in children.
Figure S2. Mediation model in which family routines and sleep environment were hypothesized to mediate associations between socioeconomic factors and sleep duration in children.
Figure S3. Higher (a) parental education and (b) family income‐to‐needs ratio were significantly associated with longer weekday sleep duration in children.
Figure S4. Higher (a) parental education and (b) family income‐to‐needs ratio were significantly associated with higher sleep environment quality.
Figure S5. More frequent family routines were significantly associated with longer weekday sleep duration in children.
Figure S6. Family routines significantly mediated the associations of (a) parental education and (b) family income‐to‐needs ratio with weekday sleep duration in children. Higher parental education and family income‐to‐needs ratio were associated with higher family routines scores which in turn were associated with longer weekday sleep duration in children. The c paths are the total effect, while the c' paths are the direct effect after accounting for the mediated effect.

## Data Availability

The data that support the findings of this study are available from the corresponding author upon reasonable request.
