# Supplementary material for: Socioeconomic disparities in sleep duration are associated with cortical thickness in children
Source: Brain Behav. 2022 Dec 27;13(2):e2859. doi: 10.1002/brb3.2859 (PMC9927856; doi:10.1002/brb3.2859)
Supplement: Supplementary file 1 — Table S1. Zero‐order correlations Figure S1. Mediation model in which sleep duration was hypothesized to mediate associations between socioeconomic factors and brain structure in children. Figure S2. Mediation model in which family routines and sleep environment were hypothesized to mediate associations between socioeconomic factors and sleep duration in children. Figure S3. Higher (a) parental education and (b) family income‐to‐needs ratio were significantly associated with longer weekday sleep duration in children. Figure S4. Higher (a) parental education and (b) family income‐to‐needs ratio were significantly associated with higher sleep environment quality. Figure S5. More frequent family routines were significantly associated with longer weekday sleep duration in children. Figure S6. Family routines significantly mediated the associations of (a) parental education and (b) family income‐to‐needs ratio with weekday sleep duration in children. Higher parental education and family income‐to‐needs ratio were associated with higher family routines scores which in turn were associated with longer weekday sleep duration in children. The c paths are the total effect, while the c' paths are the direct effect after accounting for the mediated effect. [file BRB3-13-e2859-s001.docx]

**Supplemental Material**

**Table S1**. Zero-order correlations

| **Variable** | **1** | **2** | **3** | **4** | **5** | **6** | **7** | **8** |
| --- | --- | --- | --- | --- | --- | --- | --- | --- |
| 1. Family income-to-needs ratio | -- |  |  |  |  |  |  |  |
| 2. Parental education | .67** | -- |  |  |  |  |  |  |
| 3. Sleep environment | .22* | .32** | -- |  |  |  |  |  |
| 4. Family routines | .38** | .42** | .22* | -- |  |  |  |  |
| 5. Weekday sleep duration | .22* | .27* | .06 | .38** | -- |  |  |  |
| 6. Weekend sleep duration | -.02 | .08 | .15 | .12 | .25* | -- |  |  |
| 7. Amygdala volume | .05 | .17 | .12 | .15 | .16 | -.10 | -- |  |
| 8. Hippocampal volume | .17 | .35* | -.10 | -.05 | -.19 | -.05 | .60** | -- |

*Note.* **p* < .05, ** *p* < .01


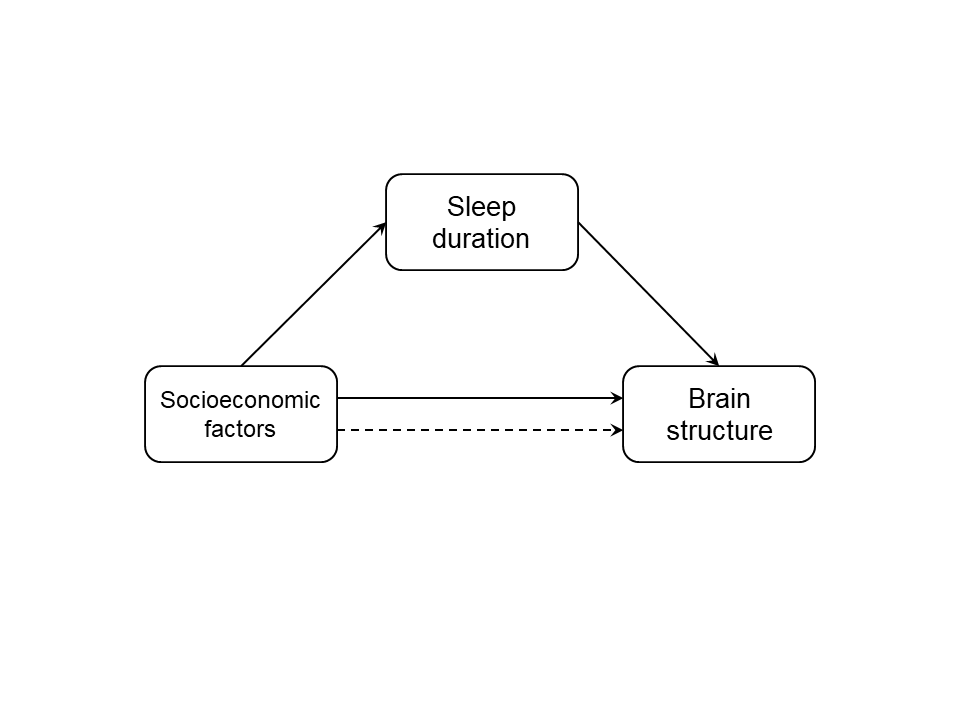


**Figure S1.** Mediation model in which sleep duration was hypothesized to mediate associations between socioeconomic factors and brain structure in children.


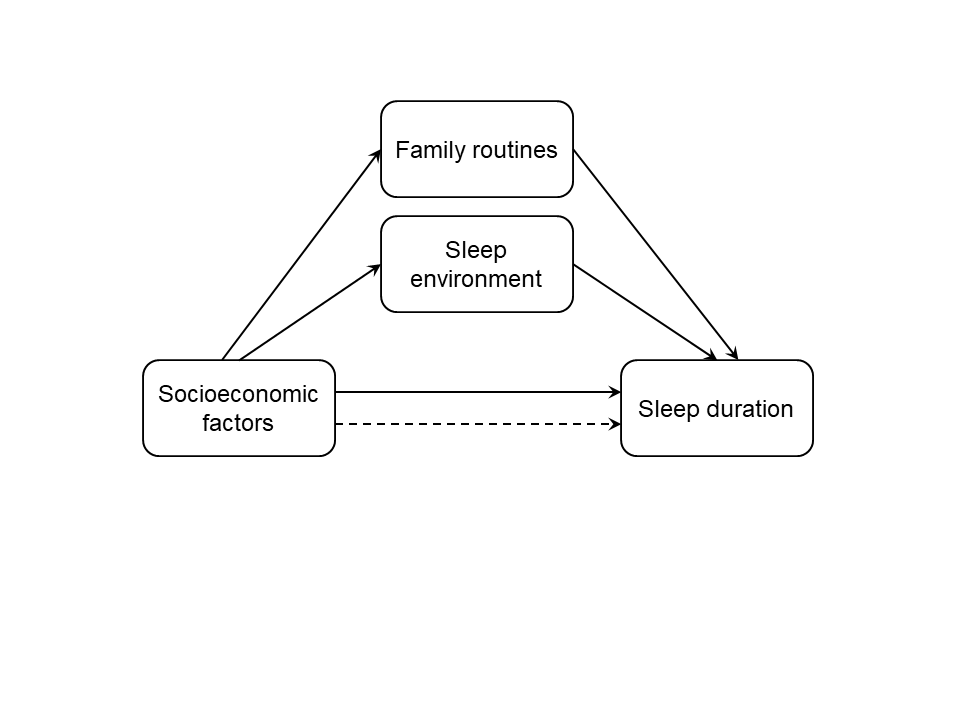


**Figure S2.** Mediation model in which family routines and sleep environment were hypothesized to mediate associations between socioeconomic factors and sleep duration in children.

| **a)** |  | **b)** |  |
| --- | --- | --- | --- |
|  | 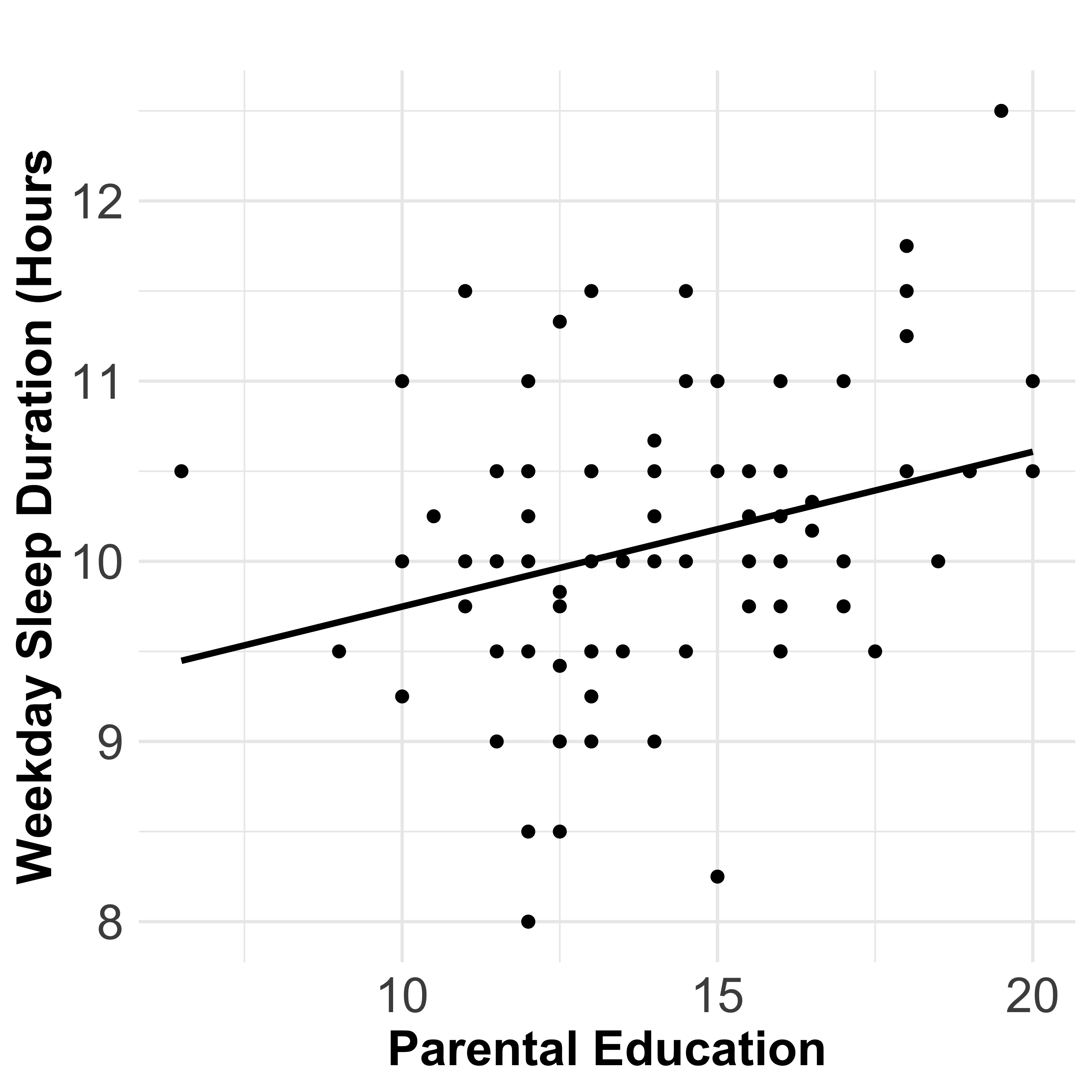 |  | 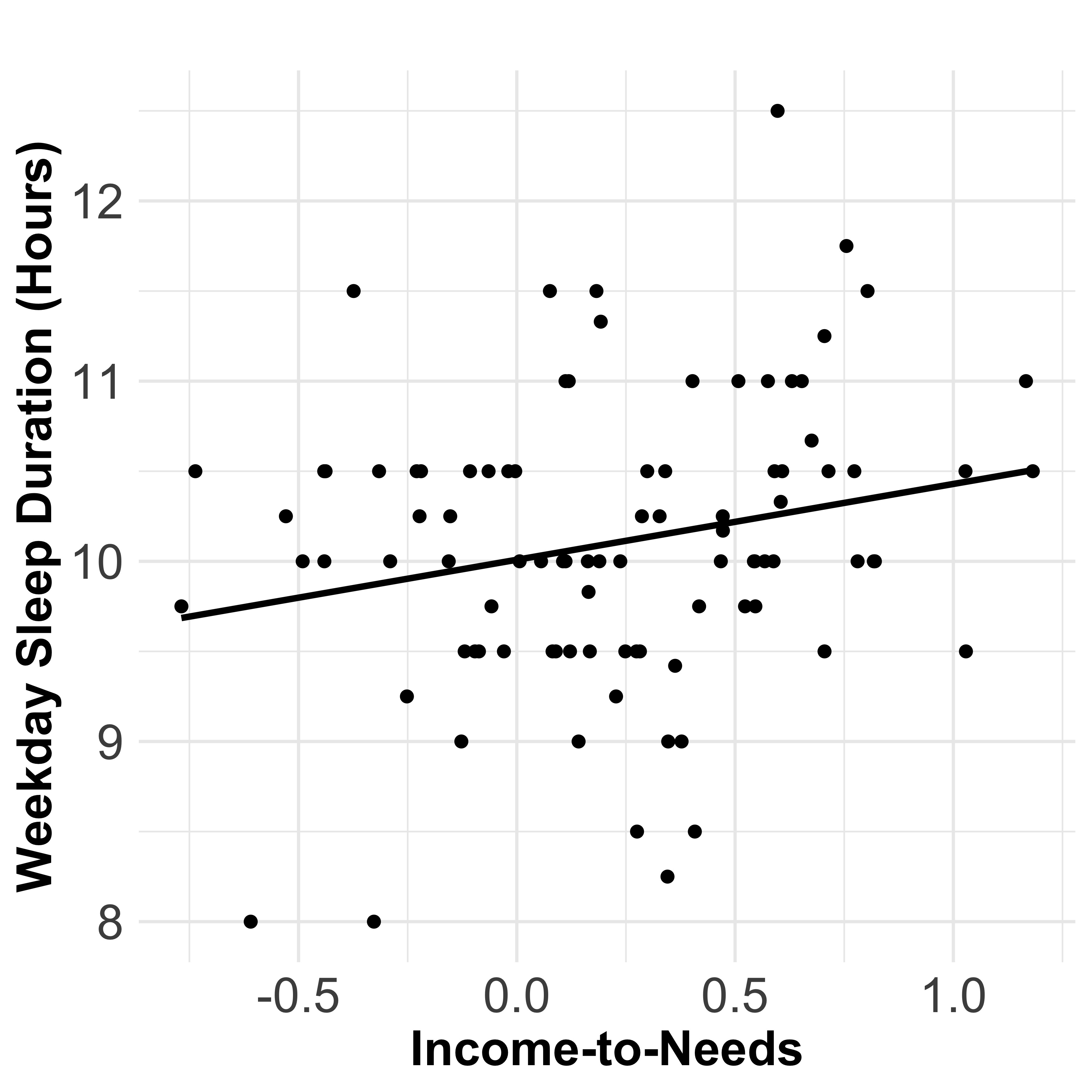 |

**Figure S3**. Higher **(a)** parental education and **(b)** family income-to-needs ratio were significantly associated with longer weekday sleep duration in children.

| **a)** |  | **b)** |  |
| --- | --- | --- | --- |
|  | 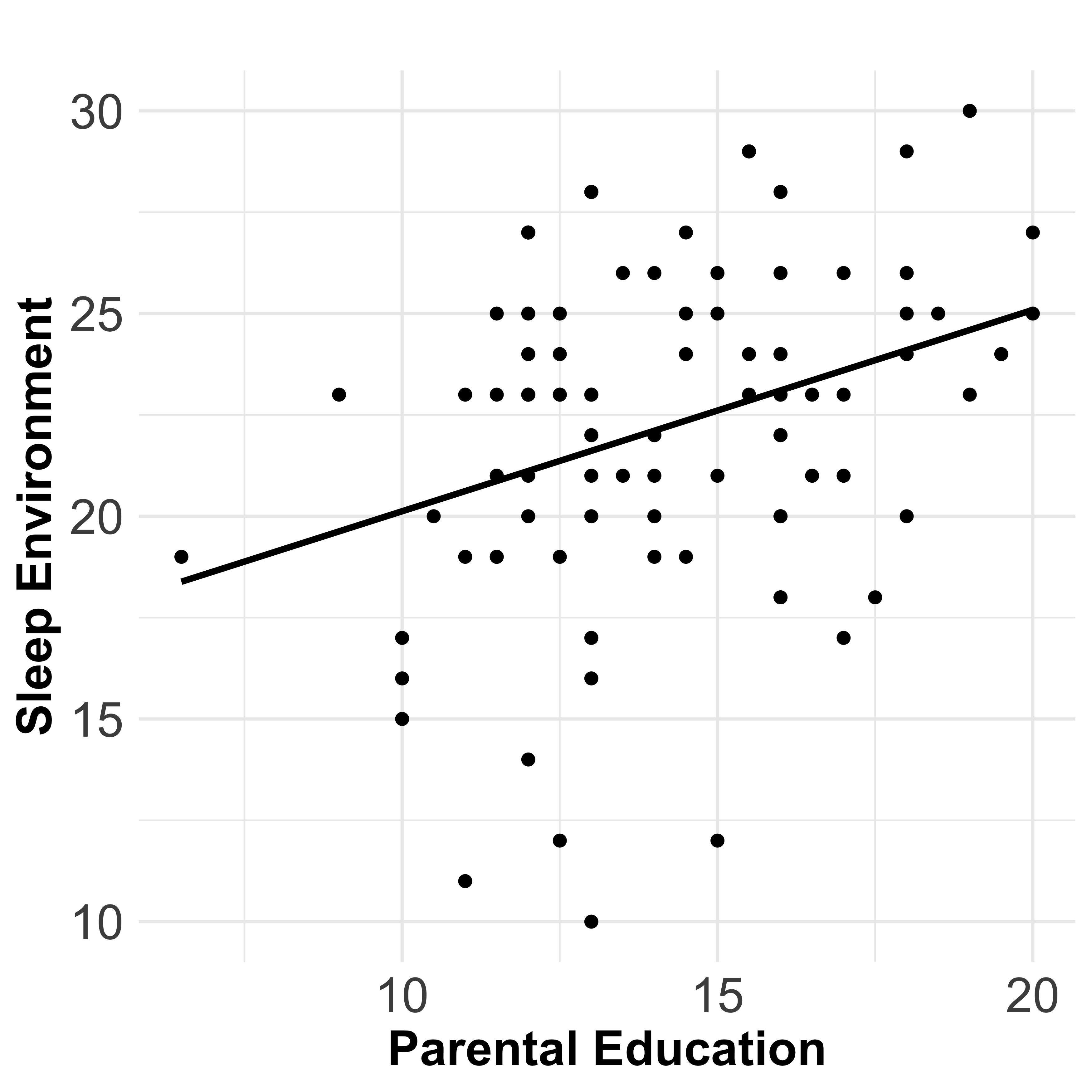 |  | 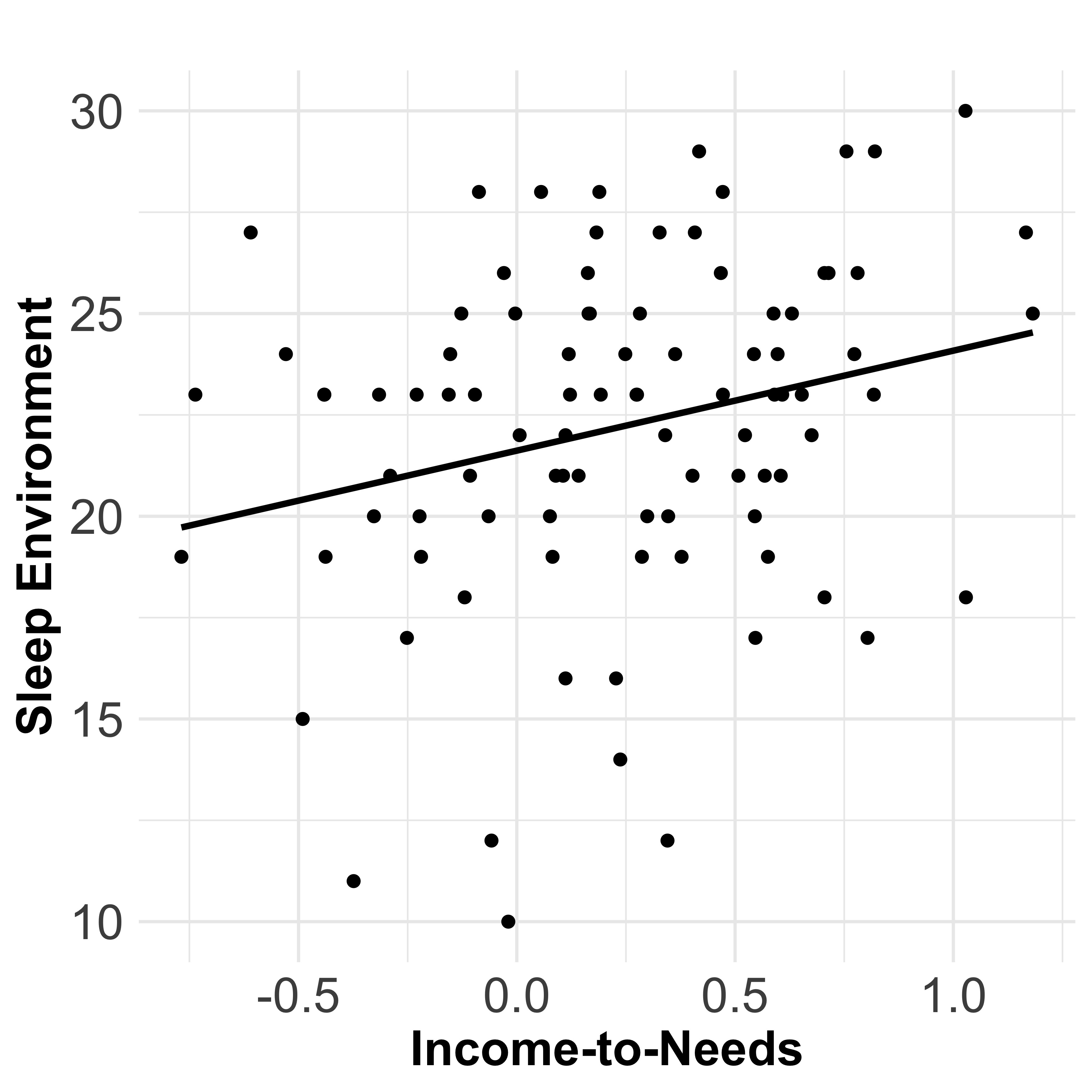 |

**Figure S4**. Higher **(a)** parental education and **(b)** family income-to-needs ratio were significantly associated with higher sleep environment quality.


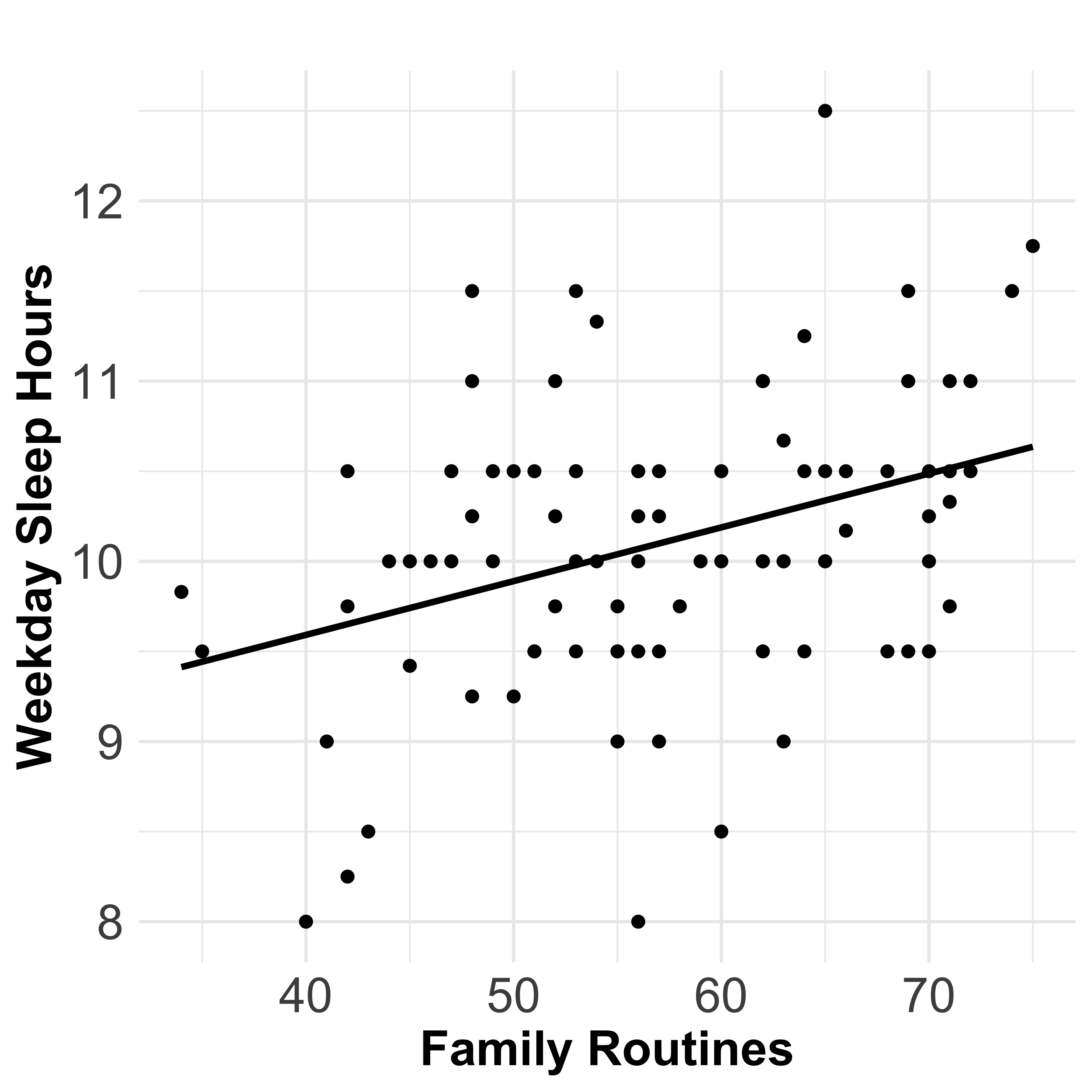


**Figure S5**. More frequent family routines were significantly associated with longer weekday sleep duration in children.

| **a)** |  | **b)** |  |
| --- | --- | --- | --- |
|  | *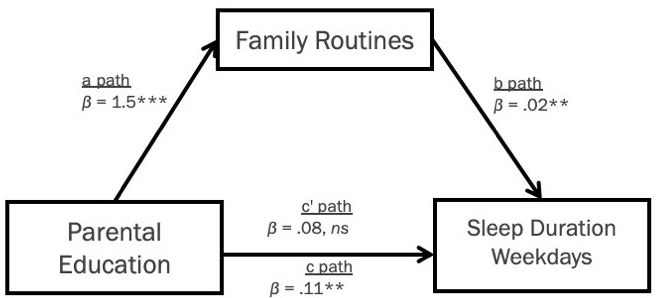* |  | *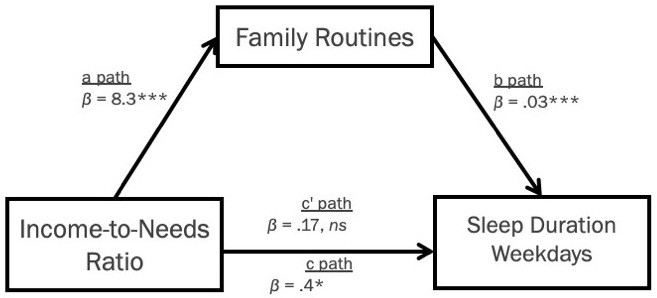* |

**Figure S6.** Family routines significantly mediated the associations of **(a)** parental education and **(b)** family income-to-needs ratio with weekday sleep duration in children. Higher parental education and family income-to-needs ratio were associated with higher family routines scores which in turn were associated with longer weekday sleep duration in children. The c paths are the total effect, while the c’ paths are the direct effect after accounting for the mediated effect.

**p* < .05, ***p* < .01, ****p* < .001, *ns* = not significant
